# Dr. Sims and the Ethics

**Published:** 1876-07

**Authors:** 


*Dr. Sims and the Ethics.*—To our friend's inquiry in regard to Dr. Sims' remarks on the ethics, we will reply that he did not denounce the ethics as a whole, but boldly objected to certain features of that time-honored code. That there are certain defects in the present system of the ethics or changes required by the different circumstances of the profession as compared with the former times we are free to admit. And we will not say that the parts to which Dr. Sims objected are not those that need revision, and if he had brought the subject properly before the body his views might have been adopted. Instead of suggesting the appointment of a committee to carefully consider the proposition and to report amendments to a subsequent meeting, he distinctly stated that he did not suggest such a committee, but clearly left the impression that if individuals of prominence and high professional standing would disregard the objectionable features and be governed by a higher law, they would be sustained.

With all deference to Dr. Sims and the high character which he has held in the profession, we frankly admit that we were shocked at his method of removing the evils which he assumed to exist, and must, therefore, demur to his remarks upon the subject. To violate a time-honored and established principle in order to sustain those who seek to make innovations upon them, even though the views suggested might not be wrong in themselves, would be manifestly hasty and impolitic, and would tend greatly to demoralize the profession and to undermine the great social and moral principles which in times past have dignified and ennobled the medical profession. The obligation to observe the ethics, therefore, still rests upon every honorable and legitimate member of the association.

As to the imputation in the letter of our correspondent in regard to using the associatiou as a medium to obtain notoriety, we can only confess that there have been those who have sought reputation and eclat by thrusting themselves forward for positions and appointments in this body.

The idea that some men can do as they please, and that others must be held to a strict adherance to the law, has given rise, in our judgment, to nearly all the troubles in the profession. It has been our misfortune to see men uphold and sustain error and wrong by dishonorable means, and afterwards declare themselves the only safe custodians of the very principles they had denounced as wrong. We fear, from the information we receive from various sections of the country, that county and State societies are often controlled by men of this character, and hence the sickly and dwarfish condition of many of these associations. It should be remembered that the means used to correct an error may be morally worse than the error sought to be removed.

And not unfrequently the men who are destitute of honor and integrity at home manage to secure high places in these Associations. Such men, however, being known at home, soon find their true level, and justly bring upon themselves the contempt and ridicule of the profession for their *vanity*, *trickery* and presumption. While, therefore, the imputation is true and is to be lamented, and has doubtless excluded many good men from this body, yet the Association should not be hastily denounced, nor the great object for which it was inaugurated be abandoned by the true men of the profession.

In the remarks we have made on this subject, we have been actuated by no ill-feeling or desire to offend the sensibilities of any one, but simply wish to avow the truth as we understand it, and to combat error from whatever source it may come.

We invite our friends to write us briefly their views touching this or other subjects, whether they agree with us or not, and we will publish them with or without their signatures, as they may direct. Our readers will doubtless appreciate truth, and correct principles whether the party uttering the same see proper to affix his signature or not.

WE contemplate, in a future number, to make further remarks upon this subject of medical ethics, and will show how it has been construed variously to suit parties in different sections of the country.

